# A Moderating Model of Self-Efficacy in Caregivers of Latina Breast Cancer Survivors

**DOI:** 10.1093/geroni/igaa057.1089

**Published:** 2020-12-16

**Authors:** Megan Thomas Hebdon, Tracy Crane, Pamela Reed, Terry Badger

**Affiliations:** 1 University of Utah, Blacksburg, Virginia, United States; 2 University of Arizona, Tucson, United States

## Abstract

In caregivers of Latina breast cancer survivors, contextual factors such as informational support, Anglo-orientation, and spiritual well-being may affect physical and mental health. The purpose of this study was to test if caregiver self-efficacy moderated relationships between contextual factors and health outcomes. A model, derived from Bandura’s Social Cognitive Theory, included self-efficacy cancer knowledge (survivor) and self-efficacy symptom management (caregiver) as moderators of relationships between contextual factors and global health and depression. Secondary analysis of baseline caregiver data from an experimental study testing two psychoeducational interventions with Latina breast cancer survivors and their caregivers was conducted. Both self-efficacy measures were tested as moderators for relationships between contextual factors and health outcomes with fixed cutoffs (medium: mean, low/high: ±1 SD). Caregiver participants (N=233) were 43 years on average (SD=13), primarily women (70%), low-income (78%), and of Mexican-American ethnicity (55%). Anglo-orientation was significantly associated with global health (r(233)=.27, p<.001) and depression (r(233)=-.13, p=.05). High levels of self-efficacy cancer knowledge strengthened the negative relationship between depression and Anglo-orientation, while a slightly positive relationship was noted at low self-efficacy levels. Informational support was significantly related to global health (r(233)=.39, p<.001) and depression (r(233)=-.43, p<.001). Self-efficacy symptom management strengthened the negative relationship between informational support and depression. Correlational and moderation relationships were not significant for spiritual well-being. Both caregiver- and survivor-focused self-efficacy affected relationships between contextual factors and depression in caregivers of Latina breast cancer survivors. Further research should address both types of self-efficacy in caregiver health.

